# Evaluation of two rapid antigen tests to detect SARS-CoV-2 in a hospital setting

**DOI:** 10.1007/s00430-020-00698-8

**Published:** 2021-01-16

**Authors:** Andreas Osterman, Hanna-Mari Baldauf, Marwa Eletreby, Jochen M. Wettengel, Suliman Q. Afridi, Thimo Fuchs, Elena Holzmann, Anton Maier, Johanna Döring, Natascha Grzimek-Koschewa, Maximilian Muenchhoff, Ulrike Protzer, Lars Kaderali, Oliver T. Keppler

**Affiliations:** 1 Max von Pettenkofer Institute and Gene Center, Virology, National Reference Center for Retroviruses, LMU München, Pettenkoferstr. 9a, 80336 Munich, Germany; 2 German Center for Infection Research (DZIF), Partner Site Munich, Munich, Germany; 3 Institute of Virology, Technical University of Munich/Helmholtz Zentrum München, Munich, Germany; 4 Institute of Bioinformatics, University Medicine Greifswald, Greifswald, Germany

**Keywords:** SARS-CoV-2 antigen test, COVID-19 point-of-care, Diagnostic test, Sensitivity, Specificity

## Abstract

Successful containment strategies for the SARS-CoV-2 pandemic will depend on reliable diagnostic assays. Point-of-care antigen tests (POCT) may provide an alternative to time-consuming PCR tests to rapidly screen for acute infections on site. Here, we evaluated two SARS-CoV-2 antigen tests: the STANDARD™ F COVID-19 Ag FIA (FIA) and the SARS-CoV-2 Rapid Antigen Test (RAT). For diagnostic assessment, we used a large set of PCR-positive and PCR-negative respiratory swabs from asymptomatic and symptomatic patients and health care workers in the setting of two University Hospitals in Munich, Germany, i.e. emergency rooms, patient care units or employee test centers. For FIA, overall clinical sensitivity and specificity were 45.4% (*n* = 381) and 97.8% (*n* = 360), respectively, and for RAT, 50.3% (*n* = 445) and 97.7% (*n* = 386), respectively. For primary diagnosis of asymptomatic and symptomatic individuals, diagnostic sensitivities were 60.9% (FIA) (*n* = 189) and 64.5% (RAT) (*n* = 256). This questions these tests’ utility for the reliable detection of acute SARS-CoV-2-infected individuals, in particular in high-risk settings. We support the proposal that convincing high-quality outcome data on the impact of false-negative and false-positive antigen test results need to be obtained in a POCT setting. Moreover, the efficacy of alternative testing strategies to complement PCR assays must be evaluated by independent laboratories, prior to widespread implementation in national and international test strategies.

## Introduction

Strategies to control the SARS-CoV-2 pandemic depend on readily available and reliable diagnostic assays to detect the virus in respiratory material. Soon after the first full-length SARS-CoV-2 genome sequence was made publicly available various RT-PCR assays were introduced [1–6]. While the overall performance of these assays is variable [5], the quantitative and sensitive detection of the SARS-CoV-2 genome by laboratory-based RT-PCR assays has undoubtedly facilitated clinical management, surveillance, contact management and disease control. However, the lack of professional laboratory staff to operate complex PCR platforms, shortages in equipment and reagents as well as the long turn-around times until test results are available, illustrate problems in a “PCR-only”-based testing strategy.

Options for additional, non-PCR-based point-of-care testing (POCT) are receiving increasing attention and are being widely implemented in national test strategies. In principle, such assays are supposed to provide rapid and reliable information on the SARS-CoV-2 infection status, e.g. in emergency departments or other health care facility settings. First published reports have stated sensitivities of SARS-CoV-2 antigen tests of 75.6% to 87.5% [7, 8].

The aim of the current study was to assess the clinical and diagnostic sensitivity and specificity of two frequently used rapid diagnostic point-of-care tests, which detect the nucleocapsid protein of SARS-CoV-2, namely the STANDARD™ F COVID-19 Ag FIA (FIA) [9] and the SARS-CoV-2 Rapid Antigen Test (RAT) [10], using a panel of PCR-positive and PCR-negative respiratory samples collected at two University Hospitals in Munich, Germany.

## Materials and methods

### Respiratory swabs

In the period March 4 to October 19, 2020, respiratory swabs (nasopharygeal or oropharyngeal) were collected by health care professionals from individuals with respiratory symptoms, who were seen in the emergency room or on clinical units of the LMU Klinikum (site 1), the second-largest University Hospital in Germany, using either the collection and transport systems eSwab™ (Copan Diagnostics, Murrieta, California, USA), ImproViral™ (Improve Medical, Guangzhou, Republic of China), dry swabs inserted into sterile 0.9% NaCl, or the original manufacturers’ swabs inserted into the extraction buffers provided, and analyzed by RT-PCR for SARS-CoV-2 RNA. Original respiratory swabs and transport media were either kept at room temperature for 1–2 h (“fresh”), stored at 4 °C for 0–7 days, or stored at − 20 °C until SARS-CoV-2 antigen testing was performed. At site 1, a total of 381 SARS-CoV-2-PCR-positive and 386 PCR-negative respiratory samples were analyzed.

At the University Hospital rechts der Isar of the Technical University of Munich (TUM), (site 2) nasopharygeal swabs were collected in the period November 13 to December 8, 2020 by health care professionals from symptomatic and asymptomatic individuals, who were seen in patient care units or the employee test center, using REST™ combi swabs (Nobel Bioscience, Sinbaek-gil, Republic of Korea) containing 2 ml universal transport medium (UTM). RT-PCR and antigen testing (RAT) were performed at the Institute of Virology on the day of submission of freshly obtained swabs. A total of 66 SARS-CoV-2-PCR-positive respiratory samples were analyzed.

### SARS-CoV-2 antigen tests

The SARS-CoV-2 Rapid Antigen Test (RAT) from Roche Diagnostics is a rapid chromatographic immunoassay intended for the qualitative, visual detection of the nucleocapsid of SARS-CoV-2 present in human nasopharynx [10]. Besides the extraction buffer provided, the manufacturer recommends the use of three specific virus transport media (VTMs) [10]. The manufacturer proposes it as a screening test in POCT settings for both symptomatic and asymptomatic individuals and states in the product sheet a test sensitivity of 96.52% and a test specificity of 99.68% based on results from studies conducted in Israel and Brazil referred to in the product sheet [10].

The SD Biosensor Standard F COVID-19 Ag FIA (FIA) is a fluorescent immunoassay for the rapid detection of SARS-CoV-2 nucleoprotein in nasopharyngeal as well as throat swabs using the STANDARD F200 Analyzer for readout [9]. The manufacturer recommends the use of eight specific VTMs [9]. The manufacturer states a sensitivity of 94% and specificity of 97% and its primary use as a screening test to aid in the early diagnosis of SARS-CoV-2 infection in patients with clinical symptoms.

Both tests were performed by laboratory personnel according to manufacturer's instructions [9, 10], unless stated otherwise. Specifically, test device and specimen were all at room temperature; equal volumes of liquid transport medium and antigen tests’ extraction buffer were mixed by vortexing or pipetting. 120 µl of this solution, corresponding to three to four drops, was applied onto the test device resulting in a complete wetting of the nitrocellulose membrane in the result’s window and subsequent visual appearance either of the control line (RAT) or disappearance of the check band when reading the setting “VTM—Group 1” (FIA). Testing was performed under a class 2 biosafety cabinet at room temperature and test devices were protected from evaporation during the incubation period. All controls integrated in the tests and quality controls for kit storage and calibration were regular. For FIA, a cutoff index (COI) ≥ 1 was interpreted as positive, for RAT every visible (even if very faint or not uniform) test line was interpreted as positive after 15 or 30 min. For FIA, the incubation was performed in a dark chamber and reading was performed after 30 min.

### Quantitative viral load determination

The following PCR assays were used for quantification in the accredited routine diagnostics laboratory of the Max von Pettenkofer Institute (site 1): the *nucleocapsid* (N1) reaction (Center for Disease Control (CDC) protocol [1], the *envelope* amplification (Charité protocol [2, 6]), the *nucleocapsid* amplification (Seegene Allplex 2019-nCoV Assay), the Roche Cobas SARS-CoV-2 *nucleocapsid* reaction or the Xpert Xpress SARS-CoV-2 run on the *GeneXpert* System. Copy number estimates were calculated as previously suggested based on the following formula [11]: ${{E}_{\mathrm{amp}}}^{(\mathrm{Intercept}-Ct)}$. The exponential amplification efficiency (*E*_amp_) and intercept (*intercept*) were derived from standard curves that were generated in multiple diluted replicates using either a plasmid containing the *nucleocapsid* gene [2019-nCoV-N-PositiveControl from IDT, a clinical sample with copy numbers based on digital droplet PCR results (site 1), as described previously [5], or an in-house *N* gene plasmid (site 2)].

At site 2, PCR assays used in the accredited routine diagnostics laboratory of the Institute of Virology (TUM) were the Real Accurate Quadruplex SARS CoV-2 PCR Kit, detecting the *N* gene and *RdRp* gene and including an inhibitory control (Pathofinder, Maastricht, Netherlands) run on a Taqman 7500 (Thermo Fisher Scientific, Waltham, USA), and the Xpert Xpress SARS-CoV-2 run on the *GeneXpert* System.

In general, the calculations for quantification do not take into account variability between separate PCR runs, different PCR chemicals or different nucleic acid extraction methods. However, since these variabilities apply to all study groups, they do not affect the interpretation of the results in this study.

## Results

### *Specificity of SARS-CoV-2 antigen tests for clinical samples is* ~ *97.7%*

To determine the specificity of both SARS-CoV-2 antigen tests, swabs taken from either the nasopharynx (*n* = 182), oropharynx (*n* = 53) or unrecorded sampling site in the upper respiratory tract (*n* = 76) derived from hospitalized adults or children at site 1 and that had been tested negative for SARS-CoV-2 RNA by RT-PCR (5), were evaluated. Both antigen tests showed a comparable specificity, 97.78% for FIA and 97.67% for RAT, with no apparent dependence on patients’ age (adults versus children) (Table 1) or sampling site (data not shown). We did not address a potential cross-reactivity with antigens from seasonal endemic beta- or alpha-coronaviruses underlying the false-positive results.

**Table 1:** Determination of assay specificity for two commercially available SARS-CoV-2 antigen tests in SARS-CoV-2 PCR-negative respiratory swabs from adults and children (< 18 years) (site 1)

Assay	Sample description	Specificity (%)	95% CI (%)	False positive/Total
STANDARD F COVID-19 Ag FIA	Adults	98.00	95.71–99.08	6/300
	Children	96.67	88.64–99.08	2/60
	Total	97.78	95.68–98.87	8/360
SARS-CoV-2 Rapid Ag Test	Adults	97.55	95.23–98.75	8/326
	Children	98.33	91.15–99.92	1/60
	Total	97.67	95.63–98.77	9/386

Binomial confidence intervals were computed using the Wilson score interval

### *The overall diagnostic sensitivity in both SARS-CoV-2 antigen tests is* ~ *50%*

**Fig. 1:**
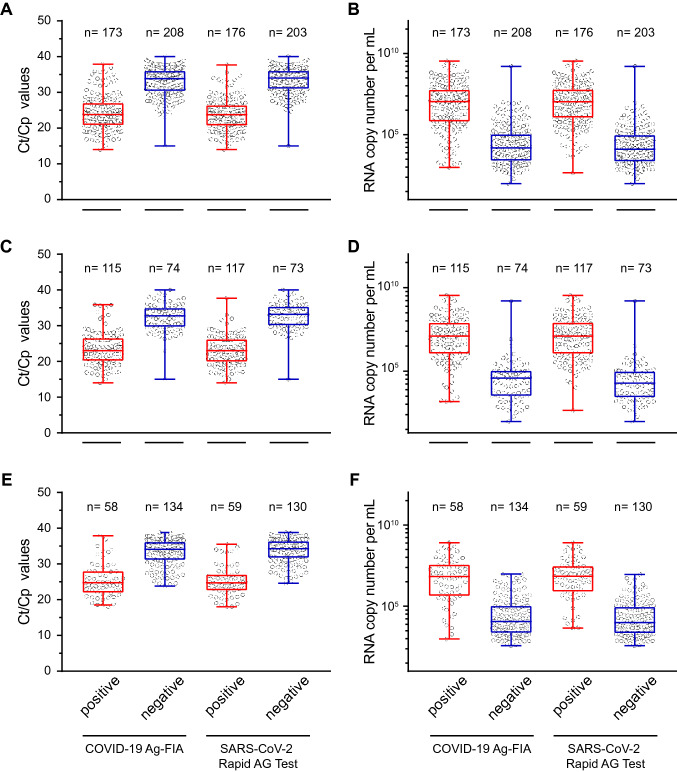
Detection of SARS-CoV-2 in 381 PCR-positive respiratory swabs from site 1 using either the STANDARD™ F COVID-19 Ag FIA or the SARS-CoV-2 Rapid Antigen Test. Respiratory swabs were analyzed and scored “positive” or “negative” according to the manufacturers’ instructions and plotted relative to either the respective sample’s *Ct*/*Cp* value (**a**) or the corresponding SARS-CoV-2 RNA copy number per mL (**b**) determined in in RT-PCR assays. Sub-analysis of swabs taken at primary diagnosis of COVID-19 (**c**, **d**) or swabs taken at follow-up testing during hospitalization (**e**, **f**). Each symbol represents one sample. Center lines show the medians and the box limits are quartiles 1 and 3, and whiskers show maximum and minimum values

Analyzing the panel of up to 445 SARS-CoV-2 RT-PCR-positive respiratory swabs from both sites, the FIA and the RAT were reactive for 45.41% (173/381) and 50.34% (224/445), respectively. For RAT, reading was performed after 15 min and after 30 min with no differences being observed for any of the specimen (data not shown). For site 1, these reactivities were plotted relative to either the *Ct*/*Cp* values (Fig. 1a) or the viral RNA copy numbers per mL (Fig. 1b) determined by SARS-CoV-2 RT-PCR in the original sample on the day of laboratory submission. Of note, both analyses are presented as complementary information since viral loads are largely independent of the PCR assay system used, yet *Ct*/*Cp* values are common practice in the literature since not all laboratories use standards for quantification.

This analysis revealed marked differences between the antigen-positive and -negative groups of specimen: the medians [lower and upper quartile] of *Ct*/*Cp* values for antigen-positive samples were 23.8 (20.9–27.0) for FIA and 23.8 (20.8–26.4) for RAT, while values for antigen-negative samples were 33.8 (30.4–36.0) for FIA and 34.0 (31.0–36.0) for RAT (Fig. 1a), respectively.

### The diagnostic sensitivity for primary diagnosis of COVID-19 based on SARS-CoV-2 antigen tests ranges between 61.6% and 72.7%

**Fig. 2:**
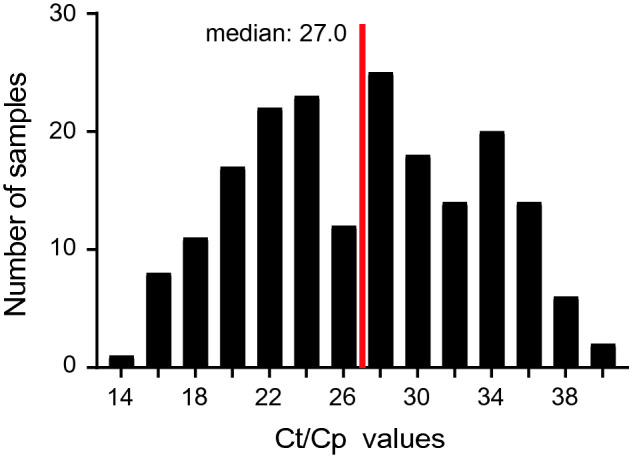
Distribution of *Ct*/*Cp* values in SARS-CoV-2 RT-PCR reactions from respiratory samples taken at primary COVID-19 diagnosis at site 1. Each bar indicates the number of respiratory samples ± 1 *Ct*/*Cp* value around the *Ct*/*Cp* value given on the *x*-axis. The red line depicts the median of these 193 specimen

Sub-analysis of respiratory samples taken from patients at “primary diagnosis” of COVID-19 in the LMU Klinikum (site 1) showed reactivity for 60.85% (115/189) for FIA (Table 2) and 61.58% (117/190) for RAT (Fig. 1c, d). A patient’s positive SARS-CoV-2 RNA detection result was classified as “primary diagnosis” when no other SARS-CoV-2 positivity had been reported prior to admission or during hospitalization. In this group, the median (lower and upper quartile) of SARS-CoV-2 RNA copies per mL in transport media of respiratory swabs for the antigen-test reactive group was 12.664.551 (1.121.832; 80.752.157). Of note, the distribution of *Ct*/*Cp* values in SARS-CoV-2 RT-PCR analyses in this important subset ranged from 14 to 40 following a near Gaussian distribution with a median *Ct*/*Cp* value of 27.0 (Fig. 2).

**Table 2:** Determination of assay sensitivity for two commercially available SARS-CoV-2 antigen tests in SARS-CoV-2 PCR-positive respiratory swabs taken at primary diagnosis (sites 1 and 2) or follow-up during hospitalization (site 1)

Assay	Sample description	Sensitivity (%)	95% CI (%)	Positive/Total
STANDARD F COVID-19 Ag FIA	Primary diagnosis	60.85	53.74–67.52	115/189
	Follow-up	30.21	24.15–37.04	58/192
	Total	45.41	40.48–50.43	173/381
SARS-CoV-2 Rapid Ag Test	Primary diagnosis	64.45	58.42–70.06	165/256
	Follow-up	31.21	25.04–38.14	59/189
	Total	50.34	45.71–54.96	224/445

Binomial confidence intervals were computed using the Wilson score interval

**Fig. 3:**
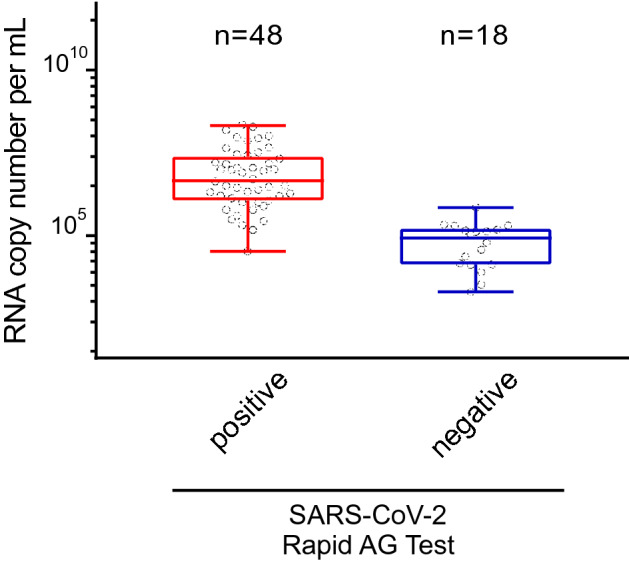
Detection of SARS-CoV-2 in 66 freshly collected, PCR-positive respiratory swabs obtained at the Klinikum rechts der Isar (site 2) and analyzed at the Institute of Virology (TUM) using the SARS-CoV-2 Rapid Antigen Test (RAT). Respiratory swabs were analyzed and scored “positive” or “negative” according to the manufacturer’s instructions and plotted relative to either the respective sample’s viral load

At the Klinikum rechts der Isar (site 2), 66 PCR-positive respiratory samples taken from symptomatic or asymptomatic individuals at primary diagnosis of COVID-19 showed reactivity for 72.73% (48/66) for RAT (Fig. 3). Here, the median (lower and upper quartile) of SARS-CoV-2 RNA copies per mL for the antigen-test reactive group was 4.545.000 (1.228.750-21.773.400).

Additional samples were analyzed that had been taken from COVID-19 patients at site 1 at “follow-up” during hospitalization, i.e. at variable time points after onset of symptoms or first PCR-positive result (Table 2; Fig. 1e, f). Here, the sensitivity dropped to 30.21% for FIA and to 31.21% for RAT with median *Ct*/*Cp* values of the samples that scored negative of 34.1 (31.2–36.1) (FIA) and 34.2 (31.8–36.3) (RAT), respectively. Many COVID-19 patients with *Ct*/*Cp* values > 30 in respiratory specimen upon admission to the hospital developed higher viral loads with lower *Ct*/*Cp* values on subsequent days (data not shown).

**Fig. 4:**
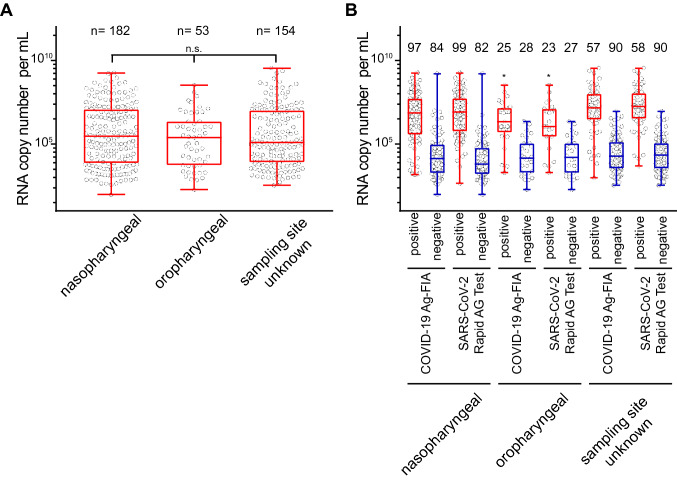
The site of swab sampling in the upper respiratory tract does not significantly affect the SARS-CoV-2 RNA load (**a**) (not significant (n.s.), *p* = 0.15, Wilcoxon rank sum test) but shows a slightly reduced antigen test reactivity for oropharyngeal swabs (**b**) (FIA: *p* = 0.029; RAT: *p* = 0.039; Fisher’s exact test) indicated by the asterix

Next, we addressed a potential influence of the sampling site for the quantitative PCR or antigen test analyses. Nasopharyngeal and oropharyngeal swabs had comparable SARS-CoV-2 viral loads (Fig. 4a). Sampling in the oropharynx, however, had a slight, but statistically significant negative effect on the reactivity of both antigen tests (FIA: *p* = 0.029; RAT: *p* = 0.039; Fisher’s exact test) (Fig. 4b). Of note, oropharyngeal swabs are commonly used in clinical practice and explicitly stated as a viable alternative for FIA, but not for RAT.

**Fig. 5:**
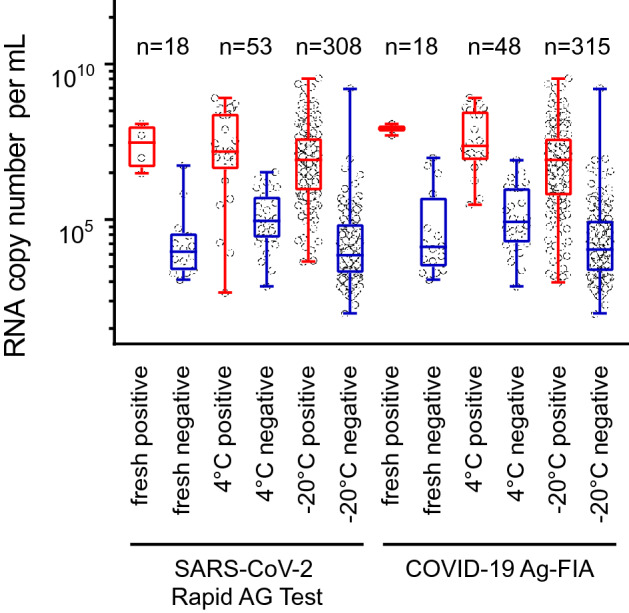
Prior storage of respiratory specimen at − 20 °C even slightly enhances the rate of SARS-CoV-2 antigen test reactivity for FIA

To rule out that the additional analysis of respiratory samples at site 1 that had been frozen at − 20 °C or stored at 4 °C may have negatively affected antigen reactivity, a comparative analysis with “fresh” [i.e. stored for 1–2 h at room temperature in the manufacturers’ extraction buffers or VTM, analogous to site 2 analyses (Fig. 3)] samples was performed. We used the former specimen to speed up the evaluation process and quickly run through a large sample set. The storage temperature of respiratory samples, either (i) “fresh”, (ii) for up to 7 days at 4 °C, or (iii) for up to 7 months at − 20 °C, did not significantly affect the antigen-positive rate for RAT with a trend towards an even lower rate for (i), the condition suggested by the manufacturers [storage group (i): 25.0%; (ii) 46.3%, (iii); 47.6%] (Fig. 5). For FIA, storage group (i) had 12.5% reactive samples only and thus a significantly lower rate (*p* = 0.013, Fisher’s exact test) than seen for group (ii) (40%) and for group (iii) (47.9%). Collectively, the additional use of stored respiratory samples in this assay validation did not negatively affect the sensitivity of either the FIA or the RAT SARS-CoV-2 antigen test.

## Discussion

Based on our results from asymptomatic and symptomatic individuals with COVID-19 seen in two major University Medical Centers in Germany, the sensitivity of the SARS-CoV-2 antigen tests evaluated was markedly lower than reported by the manufacturers. These tests’ performance for both sensitivity and specificity was inferior to the current gold standard RT-PCR and thus cannot be used interchangeably with this method to diagnose and follow COVID-19 patients or monitor early SARS-CoV-2 infection in health care workers, or for entry screening of patients in hospital or nursing home settings, as it is currently common practice in many countries.

Swab sampling in the oropharynx, rather than the nasopharynx, had a slight negative effect on the reactivity of both antigen tests, although the former sampling site has been explicitly validated for FIA according to the manufacturer. The fluorescent readout for the FIA as opposed to the visual readout for the RAT did not enhance diagnostic sensitivity (Table 2).

These antigen tests’ specificities of less than 98% may create an additional issue for their overall acceptance in the general population. Even the specificity of PCR results has recently been questioned, in particular in social media, supporting the view that independently validated performances of any SARS-CoV-2 test systems and the transparent communication of the respective results is important for continued trust into the medical and laboratory sector during this pandemic.

Swabs with high viral loads are frequently detected by both SARS-CoV-2 antigen tests. However, general claims that reactivity in an antigen test reliably identifies the group of “truly” infectious individuals or individuals with super-spreader potential under normal human interaction conditions are not substantiated by published scientific literature. Examples of an apparent super-spreader with Ct values of ≥ 27 [12] or cultivation of SARS-CoV-2 from specimen with *Ct* values ≥ 35 [13] have been reported. Moreover, recent studies estimate that around 1,000 virus particles may be sufficient for infection of a new host [14, 15], while the sensitivity of SARS-CoV-2 antigen tests ranges about 1.000-fold higher. Especially early-stage infections in our study among hospital staff at site 2 with viral loads of 10^5^ genome equivalents per mL were not detected by the POCT.

Of particular note, pre-analytical issues can negatively impact the diagnostic accuracy and affect less sensitive tests more severely [16]. To name a few of these potentially relevant pre-analytical factors: the timing of the swab relative to the onset of symptoms, the swabbing practices and test procedures, in particular when POCT is not performed by trained health care professionals. In the current study, swabs were taken exclusively by health care professionals and experienced laboratory staff conducted the assays, which positively affected the accuracy of the results. An application of these POCTs by individuals outside of the health care and laboratory sector would likely increase the risk of incorrect test results.

In addition to these caveats, it is important to consider, that diagnostic single-point measurements do not allow a reliable assessment of the ascending or descending disease state or potentially relevant clinical infectivity on the day of sampling or subsequent days in critical settings.

Similar to observations for influenza [17], the positive predictive value of antigen tests in a population with a frequency of acutely SARS-CoV-2-infected individuals of, for example, about 0.1% for Germany in mid-November 2020, is very low, i.e. ~ 2% (data derived from Tables 1, 2). Prior to RT-PCR confirmation, false-positive results may trigger inappropriate quarantine and contact tracing measures and may cause emotional distress, in particular among the elderly. While the negative predictive value of results is high (~ 99.95%), in our cohort, about 40% of SARS-CoV-2-infected and potentially infectious individuals would have been provided with a false-negative result, which may have negatively affected their own and other people’s adherence to essential protective measures. Most likely, these patients would have been admitted to non-COVID-19 wards and health care workers would have continued to work. In particular in high-risk settings, such as hospitals or elderly care facilities, the introduction of unrecognized SARS-CoV-2 may have serious adverse consequences.

Despite the advantages of rapid POCT at relatively low cost, SARS-CoV-2 antigen tests should be carried out by trained personnel and their widespread utility seems compromised by limited sensitivity and suboptimal specificity. In line with a recent editorial [18] and a comment by the Robert Koch Institute on Germany’s national testing strategy [19], we believe it is premature to advocate the widespread use of antigen-based testing in national and international strategies, as adverse consequences may outweigh benefits. We support the proposal that any new test strategy which is considered to complement current RT-PCR-centered approaches convincing high-quality outcome data, both on diagnostic accuracy and psychological impact of test results in specific environments, will be required prior to their widespread implementation.

## References

[1] Centers for Disease Control and Prevention (CDC). Information for laboratories about coronavirus (COVID-19). Atlanta: CDC. https://www.cdc.gov/coronavirus/2019-ncov/lab/index.html. Accessed 16 Apr 2020

[2] Corman VM, Landt O, Kaiser M, Molenkamp R, Meijer A, Chu DK, Bleicker T, Brünink S, Schneider J, Schmidt ML, Mulders DG, Haagmans BL, van der Veer B, van den Brink S, Wijsman L, Goderski G, Romette JL, Ellis J, Zambon M, Peiris M, Goossens H, Reusken C, Koopmans MP, Drosten C (2020). Detection of 2019 novel coronavirus (2019-nCoV) by real-time RT-PCR. Euro Surveill.

[3] van Kasteren PB, van der Veer B, van den Brink S, Wijsman L, de Jonge J, van den Brandt A, Molenkamp R, Reusken C, Meijer A (2020). Comparison of seven commercial RT-PCR diagnostic kits for COVID-19. J Clin Virol.

[4] Konrad R, Eberle U, Dangel A, Treis B, Berger A, Bengs K, Fingerle V, Liebl B, Ackermann N, Sing A (2020). Rapid establishment of laboratory diagnostics for the novel coronavirus SARS-CoV-2 in Bavaria, Germany, February 2020. Euro Surveill.

[5] Muenchhoff M, Mairhofer H, Nitschko H, Grzimek-Koschewa N, Hoffmann D, Berger A, Rabenau H, Widera M, Ackermann N, Konrad R, Zange S, Graf A, Krebs S, Blum H, Sing A, Liebl B, Wölfel R, Ciesek S, Drosten C, Protzer U, Boehm S, Keppler OT (2020). Multicentre comparison of quantitative PCR-based assays to detect SARS-CoV-2, Germany, March 2020. Euro Surveill.

[6] World Health Organization (WHO). Coronavirus disease (COVID-19) technical guidance: Laboratory testing for 2019-nCoV in humans. Geneva: WHO. https://www.who.int/emergencies/diseases/novel-coronavirus-2019/technical-guidance/laboratory-guidance. Accessed 16 Apr 2020

[7] Diao B, Wen K, Zhang J, Chen J, Han C, Chen Y, Wang S, Deng G, Zhou H, Wu Y (2020). Accuracy of a nucleocapsid protein antigen rapid test in the diagnosis of SARS-CoV-2 infection. Clin Microbiol Infect.

[8] Young S, Taylor SN, Cammarata CL, Varnado KG, Roger-Dalbert C, Montano A, Griego-Fullbright C, Burgard C, Fernandez C, Eckert K, Andrews JC, Ren H, Allen J, Ackerman R, Cooper CK (2020). Clinical evaluation of BD Veritor SARS-CoV-2 point-of-care test performance compared to PCR-based testing and versus the Sofia 2 SARS Antigen point-of-care test. J Clin Microbiol.

[9] SD Biosensor. COVID-19 Ag FIA, Package Insert. https://bestbion.com/wp-content/uploads/2020/11/bestbiondx_GA_COVID19_FIA_2020111.pdf**. **Accessed 12 Nov 2020

[10] Roche Diagnostics GmbH. SARS-CoV-2 Rapid Antigen Test, Package Insert. https://www.roche.de/res/content/11722/packungsbeilage_sars-cov-2_rapid_antigen_test.pdf. Accessed 12 Nov 2020

[11] Gallup JM (2011) Difficult templates and inhibitors of PCR. PCR troubleshooting and optimization: the essential guide. In: Kennedy S, Oswald N (eds) Caister Academic Press, UK

[12] Lin J, Yan K, Zhang J, Cai T, Zheng J (2020). A super-spreader of COVID-19 in Ningbo city in China. J Infect Public Health.

[13] Singanayagam A, Patel M, Charlett A, Lopez Bernal J, Saliba V, Ellis J, Ladhani S, Zambon M, Gopal R (2020). Duration of infectiousness and correlation with RT-PCR cycle threshold values in cases of COVID-19, England, January to May 2020. Eurosurveillance.

[14] Imai M, Iwatsuki-Horimoto K, Hatta M, Loeber S, Halfmann PJ, Nakajima N, Watanabe T, Ujie M, Takahashi K, Ito M, Yamada S, Fan S, Chiba S, Kuroda M, Guan L, Takada K, Armbrust T, Balogh A, Furusawa Y, Okuda M, Ueki H, Yasuhara A, Sakai-Tagawa Y, Lopes TJS, Kiso M, Yamayoshi S, Kinoshita N, Ohmagari N, Hattori S-i, Takeda M, Mitsuya H, Krammer F, Suzuki T, Kawaoka Y (2020). Syrian hamsters as a small animal model for SARS-CoV-2 infection and countermeasure development. Proc Natl Acad Sci.

[15] Popa A, Genger J-W, Nicholson MD, Penz T, Schmid D, Aberle SW, Agerer B, Lercher A, Endler L, Colaço H, Smyth M, Schuster M, Grau ML, Martínez-Jiménez F, Pich O, Borena W, Pawelka E, Keszei Z, Senekowitsch M, Laine J, Aberle JH, Redlberger-Fritz M, Karolyi M, Zoufaly A, Maritschnik S, Borkovec M, Hufnagl P, Nairz M, Weiss G, Wolfinger MT, von Laer D, Superti-Furga G, Lopez-Bigas N, Puchhammer-Stöckl E, Allerberger F, Michor F, Bock C, Bergthaler A (2020). Genomic epidemiology of superspreading events in Austria reveals mutational dynamics and transmission properties of SARS-CoV-2. Sci Transl Med.

[16] Payne D, Newton D, Evans P, Osman H, Baretto R (2020). Preanalytical issues affecting the diagnosis of COVID-19. J Clin Pathol.

[17] Green DA, StGeorge K (2018). Rapid antigen tests for influenza: rationale and significance of the FDA reclassification. J Clin Microbiol.

[18] Pettengill MA, McAdam AJ (2020). Can we test our way out of the COVID-19 pandemic?. J Clin Microbiol.

[19] Robert Koch Institut. Nationale Teststrategie—wer wird in Deutschland auf das Vorliegen einer SARS-CoV-2 Infektion getestet? https://www.rki.de/DE/Content/InfAZ/N/Neuartiges_Coronavirus/Teststrategie/Nat-Teststrat.html. Accessed 26 Nov 2020

